# Embryoid Body-Explant Outgrowth Cultivation from Induced Pluripotent Stem Cells in an Automated Closed Platform

**DOI:** 10.1155/2016/7098987

**Published:** 2016-08-28

**Authors:** Hiroshi Tone, Saeko Yoshioka, Hirokazu Akiyama, Akira Nishimura, Masaki Ichimura, Masaru Nakatani, Tohru Kiyono, Masashi Toyoda, Masatoshi Watanabe, Akihiro Umezawa

**Affiliations:** ^1^Department of Reproductive Biology, National Research Institute for Child Health and Development, Tokyo 157-8535, Japan; ^2^Medical Devices Division, Kaneka Corporation, Osaka 530-8288, Japan; ^3^Laboratory for Medical Engineering, Division of Materials and Chemical Engineering, Graduate School of Engineering, Yokohama National University, Kanagawa 240-8501, Japan; ^4^Medical Device Development Laboratories, Kaneka Corporation, Hyōgo 676-8688, Japan; ^5^Division of Carcinogenesis and Cancer Prevention, National Cancer Center Research Institute, Tokyo 104-0045, Japan; ^6^Research Team for Geriatric Medicine (Vascular Medicine), Tokyo Metropolitan Institute of Gerontology, Tokyo 173-0015, Japan

## Abstract

Automation of cell culture would facilitate stable cell expansion with consistent quality. In the present study, feasibility of an automated closed-cell culture system “P 4C S” for an embryoid body- (EB-) explant outgrowth culture was investigated as a model case for explant culture. After placing the induced pluripotent stem cell- (iPSC-) derived EBs into the system, the EBs successfully adhered to the culture surface and the cell outgrowth was clearly observed surrounding the adherent EBs. After confirming the outgrowth, we carried out subculture manipulation, in which the detached cells were simply dispersed by shaking the culture flask, leading to uniform cell distribution. This enabled continuous stable cell expansion, resulting in a cell yield of 3.1 × 10^7^. There was no evidence of bacterial contamination throughout the cell culture experiments. We herewith developed the automated cultivation platform for EB-explant outgrowth cells.

## 1. Introduction

Cell culture is one of the most critical bioprocesses for scientific and clinical purposes. Although cell culture has traditionally been performed manually, it presents several problems besides the risk of human error. For example, individual operational differences result in phenotypic and yield variability between different trials and institutions [1]. Furthermore, especially in clinical cell processing for cell-based therapy, manual procedures require a highly experienced staff [2], leading to higher therapeutic costs and thus preventing the widespread use of cell-based therapy [3]. Therefore, technological developments to overcome these problems are required. One possible solution is the use of an automated cell culture system.

To date, several automated cell culture systems have been reported [4–9]. Among them, the “P 4C S” (by Kaneka) [9], developed based on a prototype system [5], is a unique automated closed-culture system designed to perform all the culture manipulations in a single culture flask integrated within a single-use disposable tubing set. This system employs a unique subculture strategy which serves to limit the size of machinery and stable continual culture. However, the feasibility of this system has been shown only for bone marrow mesenchymal stromal cells and fibroblasts. For the broad range application of this system, there is a requirement to investigate the feasibility and performance of the system using many types of human cells from various tissues [10–16].

Human induced pluripotent stem cells (iPSCs) have been used for model cells of differentiation/development and diseased cells and establishment of drug screening system [17–19]. In the present study, in order to show the further applicability of “P 4C S,” we investigated the performance of this system using iPSC-derived cells and genetically immortalized keratinocytes as model cells with stable growth properties. Furthermore, we examined the applicability of this system to the EB-explant outgrowth culture as model case for explant culture.

## 2. Materials and Methods

### 2.1. Instrumentation

Cells are cultivated in “P 4C S” (Kaneka, Osaka, Japan) [9] as an enclosed system using a single-use disposable tubing set consisting of a round-shaped culture flask (surface area, 490 cm^2^), air filters, and solution bags (cell loading bag, medium bag, saline solution bag, cell detachment solution bag, cell collection bag, and waste bag). For automated cell culture, suspension of starter cells, medium, and protease (e.g., trypsin) were injected into the cell loading bag, medium bag, and cell detachment solution bag, respectively. Then, all the solution bags are connected with tubing set to form a closed circuit. The assembled tubing set is then mounted on the machinery so that the culture flask and the medium and cell detachment solution bags are separately maintained in the incubator (5% CO_2_, 37°C) and the cooler units (5°C). After cell loading into the culture flask, the system performs cell culture manipulations (medium exchange, passage, and cell harvest), whose timing program can be arbitrarily set by an operator. Here, this system performs unique passage manipulation, in which the cells are detached by trypsinization and the medium is supplied to stop the protease activity, and then the detached cells are simply dispersed uniformly by shaking flask. Following the cell dispersion, the cells were kept for short time for reattachment to the culture surface, followed by medium exchange. During the culture, fresh air (5% CO_2_) is periodically supplied to the culture flask through the air filters. In addition, images at multiple fixed positions within the culture flask are automatically captured daily by complementary metal-oxide-semiconductor camera. The detailed strategies of these manipulations are as described previously [5].

### 2.2. Ethical Statement

Studies on human cells were performed in full compliance with the Ethical Guidelines for Clinical Studies (2008 notification number 415 of the Ministry of Health, Labour, and Welfare, Japan). The cells were banked after approval of the Institutional Review Board at the National Institute of Biomedical Innovation (May 9, 2006). Animal experiments were performed according to protocols approved by the Institutional Animal Care and Use Committee of the National Research Institute for Child Health and Development.

### 2.3. Generation of iPSCs

Edom-iPS#S23 cells were generated through reprogramming by Sendai virus infection-mediated expression of OCT4, SOX2, KLF4, and c-MYC as previously described [20]. Elimination of Sendai virus was confirmed by RT-PCR. Cells just after infection served as a positive control. Sequences of the primers set are forward primer, 5′-AGA CCC TAA GAG GAC GAA GA-3′, and reverse primer, 5′-ACT CCC ATG GCG TAA CTC CAT AGT G-3′. In addition, Edom-iPS-2 cells were also established from menstrual blood-derived cells by infection with retroviruses produced from the retrovirus vector pMXs, which encodes the cDNA for human OCT3/4, SOX2, c-MYC, and KLF4 [21–24]. iPSCs were maintained on irradiated mouse embryonic fibroblasts.

### 2.4. Cell Preparation and Culture

iPSCs (MRCiPS#25) [22] were maintained on irradiated mouse embryonic fibroblasts in iPSellon medium (Cardio Incorporated, Osaka, Japan) supplemented with 1% penicillin/streptomycin solution (Life Technologies) and 10 ng/mL of basic fibroblast growth factor (bFGF; Wako, Osaka, Japan). For EB formation, iPSC colonies were mechanically cut using the STEMPRO EZPassage Tool (Life Technologies) and transferred to the low cell-adhesion 90 mm dish in iPSellon medium without bFGF. After confirming EB formation on day 3, the EBs were harvested and used for the subsequent experiment. The operation protocols were approved by the Laboratory Animal Care and the Use Committee of the National Research Institute for Child and Health Development, Tokyo.

Two types of genetically immortalized human dermal keratinocytes were used in this study. One (HDK1-K4T) was transduced with hTERT and mutant CDK4 (CDK4R24: an inhibitor resistant form of CDK4) and the other (HDK1-K4DT) was additionally transduced with cyclin D1. These genes were introduced using the recombinant lentivirus vectors by a previously described method [25]. These keratinocytes were maintained in keratinocyte-SFM supplemented epidermal growth factor, bovine pituitary extract, and 1% penicillin/streptomycin solution (all from Life Technologies) according to the manufacturer's recommendation.

### 2.5. Teratoma Formation

**Figure 1:**
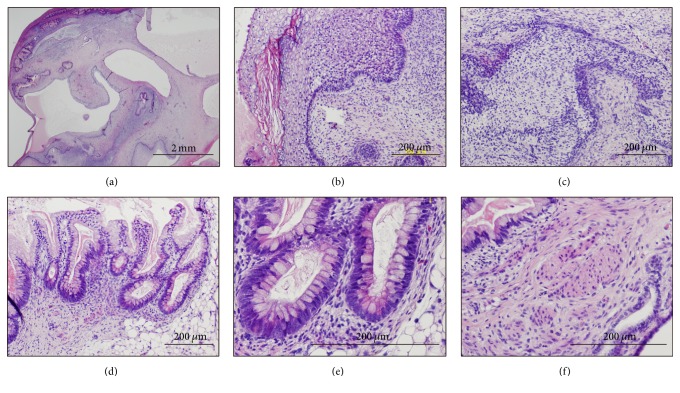
Histology of teratoma generated by Edom-iPS#S23 cells. (a) Teratoma (low-power view). (b) Epidermis. (c) Immature neuroepithelium. (d) Intestinal epithelium. (e) Intestinal epithelium with goblet and Paneth cells (high-power view of panel (d)). (f) Smooth muscle.

To address whether the Edom iPSCs have competence to differentiate into specific tissues, teratoma formation was performed by implantation of Edom iPSCs at the subcutaneous tissue (1.0 × 10^7^ cells/site) of immunodeficient mice. Edom iPSCs induced teratomas within 6–10 weeks after implantation. Histological analysis of paraffin-embedded sections demonstrated that the three primary germ layers were generated as shown by the presence of ectodermal glia and neuroepithelium, mesodermal muscle and cartilage, and endodermal ciliated epithelium morphologically in the teratoma (Figure 1).

Two types of teratoma-derived cells were used for further cultivation. One (TC1) was originally derived from the Edom-iPS-2 line (SKIP accession number SKIP000406) [22] and the other (TC2) was from Edom-iPS#S23 line (SKIP accession number SKIP000410). For cultivation of teratoma-derived cells, iPSCs were injected subcutaneously into the dorsal flank of nude mice (CLEA Japan, Japan). Three to four weeks after injection, teratomas were surgically dissected and the cells were isolated by collagenase digestion. The teratoma-dissociated cells were maintained in Dulbecco's Modified Eagle's Medium (DMEM; Life Technologies, Carlsbad, CA, USA) supplemented with 10% fetal bovine serum (FBS) and 1% penicillin/streptomycin solution (Life Technologies). The operation protocols were approved by the Laboratory Animal Care and Use Committee of the National Research Institute for Child and Health Development, Tokyo.

### 2.6. Automated Cell Culture

For teratoma-derived cell and keratinocyte cultures, 3.0 × 10^6^ (teratoma-derived cells) and 1.5–1.8 × 10^7^ cells (keratinocytes) were placed into the system and cultured using the media described above. The medium exchanges were performed twice a week.

For EB-explant outgrowth culture, iPSCs were dissociated into single cells with accutase (Thermo Scientific, MA, USA) after exposure to the rock inhibitor (Y-27632: A11105-01, Wako, Japan) and then passaged into the 90 mm dishes coated with 0.1% gelatin solution (Sigma-Aldrich, St. Louis, MO, USA) at a density of 10,000 cells/dish in the EB medium containing 76% Knockout DMEM, 20% Knockout Serum Replacement (Life Technologies, CA, USA), 2 mM GlutaMAX-I, 0.1 mM NEAA, Pen-Strep, and 50 *μ*g/mL L-ascorbic acid 2-phosphate (Sigma-Aldrich, St. Louis, MO, USA). Thereafter, the EBs collected from two dishes were placed into the system and cultured using DMEM/Nutrient Mixture F-12 (Life Technologies) supplemented with 20% FBS, 1% Pen-Strep solution, 1% NEAA (Life Technologies), 1% sodium pyruvate solution (Life Technologies), and GlutaMAX supplement (Life Technologies). The medium exchanges were performed twice a week. When sufficient cell outgrowth from EBs was observed, the cells were passaged (on day 7), and the cultivation was continued further.

### 2.7. Analysis after Automated Cell Culture

For cell harvesting, the culture medium was automatically removed from the flask. The cells were washed with saline twice to remove cell debris and the remaining medium and were scraped by adding trypsin-EDTA solution to the culture flask. The flask was incubated in the incubator at 37°C for 3–10 min. The flask was gently swung for cell removal from the surface. The cell numbers and viabilities were measured using a Vi-Cell XR cell viability analyzer (Beckman Coulter, Brea, CA, USA). The cells after the propagation with the automated cultivation system were also applied to gene chip analysis for the postprocess validation.

## 3. Results and Discussion

**Figure 2:**
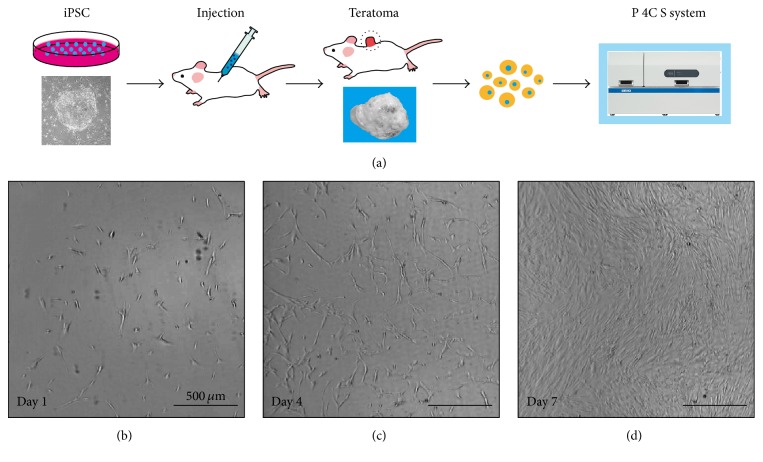
Time-course images of teratoma-derived cells in automated culture. (a) Scheme for automated culture of teratoma-derived cells. ((b), (c), (d)) Phase-contrast photomicrography of day 1 (b), day 4 (c), and day 7 (d) after the start of the automated culture. The experiments were repeated four independent times. The scale bars indicate 500 *μ*m.

**Figure 3:**
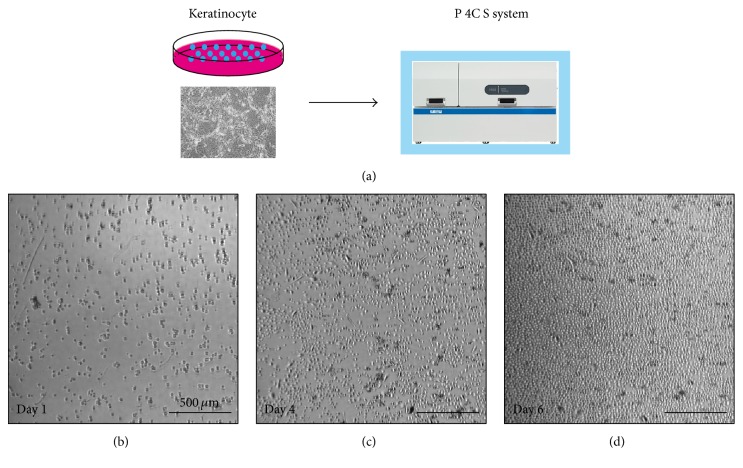
Time-course images of keratinocytes in automated culture. (a) Scheme for automated culture of keratinocytes. ((b), (c), (d)) Phase-contrast photomicrography of day 1 (b), day 4 (c), and day 6 (d) after the start of the automated culture. The experiments were repeated three independent times. The scale bars indicate 500 *μ*m.

First, we examined the performance of “P 4C S” using teratoma-derived cells (TC1 and TC2) and genetically immortalized keratinocytes (HDK1-K4T and HDK1-K4DT) as model cells. On day 1 in each culture, we confirmed that all of the cells were distributed uniformly throughout the culture flask by automatically obtained images at multiple fixed positions, which is a key factor for stable cell growth. The cell distribution is probably due to the P 4C S's specialized design where the starter cells are dispersed by shaking the culture flask, based on the optimized particle dispersion simulation [5]. TC1 and HDK1-K4DT cells were stably expanded depending on the culture duration (Figures 2 and 3). iPSC-teratoma-derived cells and keratinocytes were successfully expanded to more than 4.5 × 10^7^ cells (a 15-fold increase over 7 days) and 5.1 × 10^7^ cells (3.4-fold increase for 6 days) with high viabilities, respectively (Tables 1 and 2). The antibiotics were used for teratoma-derived cells since bacterial contamination cannot be eliminated. The media fill test revealed that the automated cell cultivation system used in this study is able to produce cells without microbial contamination [26].

**Figure 4:**
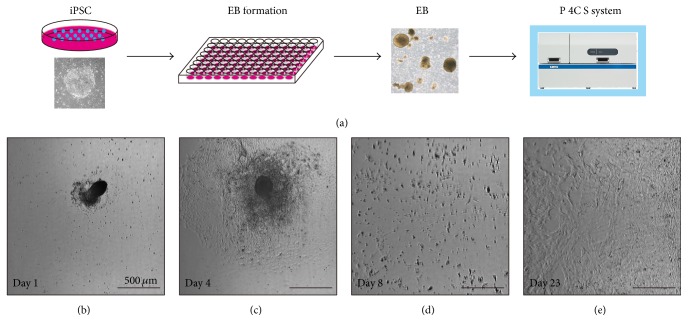
Time-course images of iPSC-derived EB-explant outgrowth cells in automated culture. (a) Scheme for automated culture of EB-explant outgrowth cells. ((b), (c), (d), (e)) Phase-contrast photomicrography of day 1 (b), day 4 (c), day 8 (d), and day 23 (e) after the start of the automated culture. The EBs adhered to the culture surface (on day 1) and the cell outgrowth was clearly confirmed surrounding the EBs (on day 4), after which we performed the subculture manipulation (on day 7). The uniform cell distribution after passage could be confirmed by an image on day 8. The cells were cultured until reaching subconfluent state (on day 23). The experiments were repeated three independent times. The scale bars indicate 500 *μ*m.

EB formation from embryonic stem cells (ESCs) or iPSCs and following EB-explant outgrowth culture is a common strategy for generation of different cell lineages and expansion of differentiated cells for further applications [27–31]. In this study, we examined the feasibility of this system for the iPSC-derived EB-explant outgrowth culture as a model case for explant culture. iPSC-derived EBs successfully adhered to the culture surface (on day 1) and the cell outgrowth was clearly confirmed (on day 4), while the cells were only found surrounding the adherent EBs (Figure 4). After confirming cell outgrowth, we detached and dispersed the cells by shaking the culture flask, leading to uniform cell distribution (on day 8). After the subculture, the cells were stably expanded evenly throughout the culture flask and reached subconfluent state on day 23, resulting in cell yields of 3.1 × 10^7^ cells (Table 3). Also, we confirmed that this culture strategy was applicable for human ESC-derived EBs and that the resultant cell yield was similar to that obtained from iPSC-derived EBs. Actually, creation of uniform cell distribution is essential for continuous stable expansion culture [26]. Thus, this strategy may be also useful for other explant cultures including mesenchymal stem cells [32, 33], keratinocytes [34], and fibroblasts [35], which have been widely used for regenerative medicine purposes.

Conventionally, cell cultures have been performed using open culture vessels which are vulnerable to bacterial contamination. This is an important concern in both clinical and scientific settings. For clinical cell processing in cell-based therapy, to minimize the risk of contamination, a clean facility is indispensable and its cleanliness is strictly maintained by several means such as air-conditioning, differential pressure, and various sanitary controls [36, 37]. However, the requirement of such facility drives the therapeutic cost up, which has severely hampered the spread of cell-based therapy. In contrast to the open culture vessels, theoretically, the use of “P 4C S” that employs closed-culture vessel does not require a clean environment. In fact, we performed cell culture experiments with the machine located in a conventional laboratory, resulting in no evidence of bacterial contamination. Therefore, the installation of “P 4C S” into clinical cell processing has huge advantages not only for cell culture automation but also for reduced requirement of a clean facility, which would facilitate the wide spread of cell-based therapy.

## 4. Conclusions

We show here the applicability and performance of “P 4C S” in teratoma-derived cells, keratinocytes, and EB outgrowth cultures. It is noteworthy that the “P 4C S” specific subculture manipulation enables creation of uniform cell distribution which was useful for EB-explant outgrowth cultures for continual stable expansion. In addition, all culture experiments in this study could be performed without bacterial contamination. These results suggest that the use of “P 4C S” is a promising approach to overcome the problems in current manual procedure for clinical and scientific purposes.

## Figures and Tables

**Table 1:** Automated culture of teratoma-derived cells.

	Experiment 1	Experiment 2
Cell	TC1	TC2
Culture period	7 days	7 days
Cell number		
Seeded	0.3 × 10^7^ cells	0.3 × 10^7^ cells
Harvested	5.8 × 10^7^ cells	4.5 × 10^7^ cells
Fold increase	19-fold	15-fold
Cell viability	93%	93%

**Table 2:** Automated culture of keratinocytes.

	Experiment 1	Experiment 2
Cell	HDK1-K4T	HDK1-K4DT
Culture period	6 days	6 days
Cell number		
Seeded	1.8 × 10^7^ cells	1.5 × 10^7^ cells
Harvested	5.4 × 10^7^ cells	5.1 × 10^7^ cells
Fold increase	3.0-fold	3.4-fold
Cell viability	95%	93%

**Table 3:** Automated EB-explant outgrowth culture.

	iPSC-derived EBs
Culture period	23 days
Cell number	
Seeded	Not calculated
Harvested	3.1 × 10^7^ cells
Cell viability	84%

## References

[1] Tran C. A., Burton L., Russom D. (2007). Manufacturing of large numbers of patient-specific T cells for adoptive immunotherapy: an approach to improving product safety, composition, and production capacity. *Journal of Immunotherapy*.

[2] Mason C., Hoare M. (2007). Regenerative medicine bioprocessing: building a conceptual framework based on early studies. *Tissue Engineering*.

[3] Okano T., Sawa Y., Barber E., Umezawa A. (2015). Regenerative therapy by fusion of medicine and engineering: first-in-human clinical trials with induced pluripotent stem cells and cell sheet technology: a report of the Symposium of Regenerative Medicine for Patients. *Regenerative Therapy*.

[4] Kami D., Watakabe K., Yamazaki-Inoue M. (2013). Large-scale cell production of stem cells for clinical application using the automated cell processing machine. *BMC Biotechnology*.

[5] Kato R., Iejima D., Agata H. (2010). A compact, automated cell culture system for clinical scale cell expansion from primary tissues. *Tissue Engineering Part C: Methods*.

[6] Kobayashi T., Kan K., Nishida K., Yamato M., Okano T. (2013). Corneal regeneration by transplantation of corneal epithelial cell sheets fabricated with automated cell culture system in rabbit model. *Biomaterials*.

[7] Rojewski M. T., Fekete N., Baila S. (2013). GMP-compliant isolation and expansion of bone marrow-derived MSCs in the closed, automated device quantum cell expansion system. *Cell Transplantation*.

[8] Thomas R. J., Anderson D., Chandra A. (2009). Automated, scalable culture of human embryonic stem cells in feeder-free conditions. *Biotechnology and Bioengineering*.

[9] Akiyama H., Kobayashi A., Ichimura M. (2015). Comparison of manual and automated cultures of bone marrow stromal cells for bone tissue engineering. *Journal of Bioscience and Bioengineering*.

[10] Xie L., Yang R., Liu S. (2016). TR3 is preferentially expressed by bulge epithelial stem cells in human hair follicles. *Laboratory Investigation*.

[11] Matsushita K., Morello F., Zhang Z. (2016). Nuclear hormone receptor LXR*α* inhibits adipocyte differentiation of mesenchymal stem cells with Wnt/beta-catenin signaling. *Laboratory Investigation*.

[12] Kameishi S., Sugiyama H., Yamato M. (2015). Remodeling of epithelial cells and basement membranes in a corneal deficiency model with long-term follow-up. *Laboratory Investigation*.

[13] Tanaka K., Fujita T., Umezawa H. (2014). Therapeutic effect of lung mixed culture-derived epithelial cells on lung fibrosis. *Laboratory Investigation*.

[14] Abe-Suzuki S., Kurata M., Abe S. (2014). CXCL12+ stromal cells as bone marrow niche for CD34+ hematopoietic cells and their association with disease progression in myelodysplastic syndromes. *Laboratory Investigation*.

[15] Pirraco R. P., Iwata T., Yoshida T. (2014). Endothelial cells enhance the *in vivo* bone-forming ability of osteogenic cell sheets. *Laboratory Investigation*.

[16] Takano T., Li Y.-J., Kukita A. (2014). Mesenchymal stem cells markedly suppress inflammatory bone destruction in rats with adjuvant-induced arthritis. *Laboratory Investigation*.

[17] Santostefano K. E., Hamazaki T., Biel N. M., Jin S., Umezawa A., Terada N. (2015). A practical guide to induced pluripotent stem cell research using patient samples. *Laboratory Investigation*.

[18] De Assuncao T. M., Sun Y., Jalan-Sakrikar N. (2015). Development and characterization of human-induced pluripotent stem cell-derived cholangiocytes. *Laboratory Investigation*.

[19] Higuchi A., Ling Q., Kumar S. S. (2014). Generation of pluripotent stem cells without the use of genetic material. *Laboratory Investigation*.

[20] Nishimura K., Sano M., Ohtaka M. (2011). Development of defective and persistent Sendai virus vector: a unique gene delivery/expression system ideal for cell reprogramming. *The Journal of Biological Chemistry*.

[21] Makino H., Toyoda M., Matsumoto K. (2009). Mesenchymal to embryonic incomplete transition of human cells by chimeric OCT4/3 (POU5F1) with physiological co-activator EWS. *Experimental Cell Research*.

[22] Nishino K., Toyoda M., Yamazaki-Inoue M. (2011). DNA methylation dynamics in human induced pluripotent stem cells over time. *PLoS Genetics*.

[23] Cui C.-H., Uyama T., Miyado K. (2007). Menstrual blood-derived cells confer human dystrophin expression in the murine model of Duchenne muscular dystrophy via cell fusion and myogenic transdifferentiation. *Molecular Biology of the Cell*.

[24] Hida N., Nishiyama N., Miyoshi S. (2008). Novel cardiac precursor-like cells from human menstrual blood-derived mesenchymal cells. *STEM CELLS*.

[25] Sasaki R., Narisawa-Saito M., Yugawa T. (2009). Oncogenic transformation of human ovarian surface epithelial cells with defined cellular oncogenes. *Carcinogenesis*.

[26] Akiyama H., Kobayashi A., Ichimura M. (2015). Comparison of manual and automated cultures of bone marrow stromal cells for bone tissue engineering. *Journal of Bioscience and Bioengineering*.

[27] Mahmood A., Harkness L., Schrøder H. D., Abdallah B. M., Kassem M. (2010). Enhanced differentiation of human embryonic stem cells to mesenchymal progenitors by inhibition of TGF-*β*/activin/nodal signaling using SB-431542. *Journal of Bone and Mineral Research*.

[28] Sasaki N., Hirano T., Kobayashi K. (2010). Chemical inhibition of sulfation accelerates neural differentiation of mouse embryonic stem cells and human induced pluripotent stem cells. *Biochemical and Biophysical Research Communications*.

[29] Taura D., Noguchi M., Sone M. (2009). Adipogenic differentiation of human induced pluripotent stem cells: comparison with that of human embryonic stem cells. *FEBS Letters*.

[30] Takahashi K., Narita M., Yokura M., Ichisaka T., Yamanaka S. (2009). Human induced pluripotent stem cells on autologous feeders. *PLoS ONE*.

[31] Masuda S., Matsuura K., Anazawa M., Iwamiya T., Shimizu T., Okano T. (2015). Formation of vascular network structures within cardiac cell sheets from mouse embryonic stem cells. *Regenerative Therapy*.

[32] Mori Y., Ohshimo J., Shimazu T. (2015). Improved explant method to isolate umbilical cord-derived mesenchymal stem cells and their immunosuppressive properties. *Tissue Engineering Part C: Methods*.

[33] Yoon J. H., Roh E. Y., Shin S. (2013). Comparison of explant-derived and enzymatic digestion-derived MSCs and the growth factors from Wharton's jelly. *BioMed Research International*.

[34] Guo A., Jahoda C. A. (2009). An improved method of human keratinocyte culture from skin explants: cell expansion is linked to markers of activated progenitor cells. *Experimental Dermatology*.

[35] Watson D., Keller G. S., Lacombe V., Fodor P. B., Rawnsley J., Lask G. P. (1999). Autologous fibroblasts for treatment of facial rhytids and dermal depressions. A pilot study. *Archives of Facial Plastic Surgery*.

[36] Owaki T., Shimizu T., Yamato M., Okano T. (2014). Cell sheet engineering for regenerative medicine: current challenges and strategies. *Biotechnology Journal*.

[37] Hayakawa T., Aoi T., Umezawa A. (2015). A study on ensuring the quality and safety of pharmaceuticals and medical devices derived from processing of autologous human induced pluripotent stem(-like) cells. *Regenerative Therapy*.

